# Genetic Variation in the Extended Major Histocompatibility Complex and Susceptibility to Childhood Acute Lymphoblastic Leukemia: A Review of the Evidence

**DOI:** 10.3389/fonc.2013.00300

**Published:** 2013-12-12

**Authors:** Kevin Y. Urayama, Pamela D. Thompson, Malcolm Taylor, Elizabeth A. Trachtenberg, Anand P. Chokkalingam

**Affiliations:** ^1^School of Public Health, University of California, Berkeley, CA, USA; ^2^Center for Clinical Epidemiology, St. Luke’s Life Science Institute, Tokyo, Japan; ^3^Cancer Immunogenetics, St. Mary’s Hospital, University of Manchester, Manchester, UK; ^4^Handforth, Cheshire, UK; ^5^Center for Genetics, Children’s Hospital Oakland Research Institute, Oakland, CA, USA

**Keywords:** childhood leukemia, epidemiology, genetic susceptibility, human leukocyte antigen, major histocompatibility complex

## Abstract

The enduring suspicion that infections and immunologic response may play a role in the etiology of childhood leukemia, particularly acute lymphoblastic leukemia (ALL), is now supported, albeit still indirectly, by numerous epidemiological studies. The cumulative evidence includes, for example, descriptive observations of a peculiar peak incidence at age 2–5 years for ALL in economically developed countries, clustering of cases in situations of population mixing associated with unusual patterns of personal contacts, associations with various proxy measures for immune modulatory exposures early in life, and genetic susceptibility conferred by variation in genes involved in the immune system. In this review, our focus is the extended major histocompatibility complex (MHC), an approximately 7.6 Mb region that is well-known for its high-density of expressed genes, extensive polymorphisms exhibiting complex linkage disequilibrium patterns, and its disproportionately large number of immune-related genes, including human leukocyte antigen (HLA). First discovered through the role they play in transplant rejection, the classical HLA class I (HLA-A, -B, and -C) and class II (HLA-DR, HLA-DQ, and HLA-DP) molecules reside at the epicenter of the immune response pathways and are now the targets of many disease susceptibility studies, including those for childhood leukemia. The genes encoding the HLA molecules are only a minority of the over 250 expressed genes in the xMHC, and a growing number of studies are beginning to evaluate other loci through targeted investigations or utilizing a mapping approach with a comprehensive screen of the entire region. Here, we review the current epidemiologic evidence available to date regarding genetic variation contained within this highly unique region of the genome and its relationship with childhood ALL risk.

## Introduction

Leukemia, a cancer of the hematopoietic system, is the most common malignancy affecting children less than 15 years of age. Acute lymphoblastic leukemia (ALL) comprises nearly 80% of childhood leukemia diagnoses in developed countries, followed by acute myeloid leukemia (16%) and the very rare chronic sub-types (1, 2). Pediatric patients usually exhibit characteristic chromosomal aberrations such as hyperdiploidy and/or translocations [e.g., *t*(12;21) *TEL-AML1*, 11q23*MLL-AF4, t*(8;21) *AML1-ETO, t*(15;17) *PML-RARA*, inv(16) *CPFB-MYH11*] (3), most of which have been shown to originate prenatally due to unknown causes (2). These prenatal chromosomal aberrations are likely initiating genetic events, occurring during fetal hematopoiesis and operating within a minimal two-hit disease model. Current evidence indicates that transition to overt disease will occur in only a small proportion of children carrying this pre-leukemic clone, after a sufficient second genetic event which likely occurs postnatally as a consequence of a mutagenic exposure (2, 4).

One potential post-natal exposure long thought to modulate this second “hit” is exposure to infectious agents, a premise based on early studies demonstrating the ability of certain viruses to cause leukemia in animals (5, 6) and observing an age distribution that closely mimics the incidence of common childhood infectious diseases (7). In the absence of direct evidence for the involvement of a specific infection (8), this immunologic hypothesis describing a role of infections in childhood leukemia, particularly B-cell precursor (BCP) ALL, has persisted as a result of accumulating epidemiological evidence largely driven by two related infectious hypotheses proposed in the late 1980s. Kinlen proposed the “population mixing” hypothesis in response to childhood leukemia clusters occurring in Seascale and Thurso (UK), and explained that the rise in childhood leukemia incidence may have resulted from children experiencing an abnormal immune response to specific infections introduced during an influx of infected persons into an area populated with non-immune, thus susceptible individuals (9). Greaves posited his “delayed infection” hypothesis in the context of the two-hit disease model, describing that the second genetic hit leading to overt disease in a small fraction (∼1%) of pre-leukemia carriers may be caused by an adverse immune response to infections as a result of insufficient priming of the immune system in early life (10). Abundant epidemiological data are currently available in support of both the population mixing (11) and delayed infection hypotheses (12). Furthermore, new evidence has emerged suggesting that immune dysregulation caused by an infectious exposure in the first months of life may lead to an increased risk of ALL in a genetically susceptible subset of children (2, 13).

**Figure 1:**
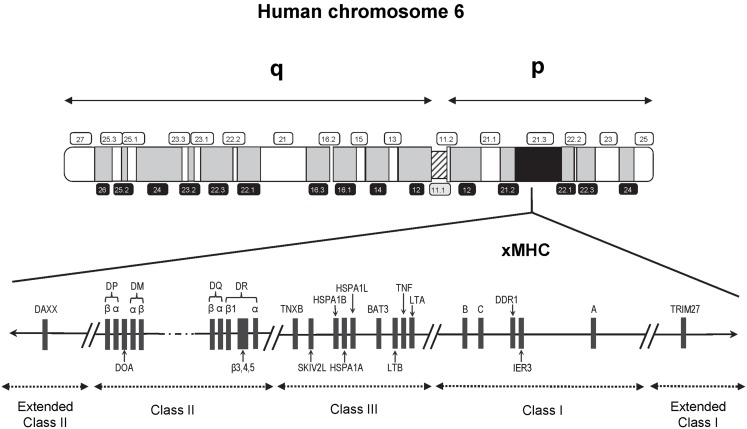
**Schematic map (not to scale) of the 7.6 Mb extended human MHC (xMHC) on the short (p) arm of human chromosome 6**. The HLA class I gene cluster includes the three expressed classical class I genes, *HLA-A, HLA-B*, and *HLA-C*, and is located telomeric of the class III region. The HLA class II gene cluster is located centromeric of the HLA class III region. Of the over 250 known expressed loci of the xMHC, the relative locations of only those that have appeared in previous studies of childhood ALL are indicated.

Findings from genetic susceptibility studies targeting immune-related genetic loci have also fueled an ongoing suspicion of a role for immunologic response involving infections. Foremost among the candidate loci are those residing within the major histocompatibility complex (MHC), a genomic region well-known for its high-density of genes, complex linkage disequilibrium (LD), and highly polymorphic nature. The human MHC region spans approximately 4 Mb on the short arm of chromosome 6 (6p21.3) and contains about 250 gene loci (Figure 1), with a disproportionately large number encoding genes that play a role in immune function and regulation (14). This region first received attention following the discovery of cell surface molecules called the human leukocyte antigens (HLA) involved in tissue graft and organ transplant rejection. HLA is now well-understood to have a primary functional role in the protection against pathogens through selective binding and presentation, in allele selective fashion, of processed peptides to T lymphocytes critical to both cellular and humoral immune responses.

Recent interrogation of the flanking regions led to the discovery of additional MHC-relevant genes that extended beyond the classically defined boundaries (15). This expansion resulted in a region that is now referred to as the extended MHC (xMHC) spanning 7.6 Mb and is divided into five subregions consisting of the extended class I region at the telomeric end, and successively the classical class I, III, and II clusters bounded by an extended class II region at the centromeric end (Figure 1). While the HLA superfamily of genes are among the most highly implicated targets in disease susceptibility, they account for only a small proportion of the estimated 252 expressed xMHC loci (15), including genes involved in other immune response and inflammatory processes (e.g., cytokines and complement factors), leukocyte maturation, stress response, cell-cycle regulation, transcriptional and translational machinery, and other cellular processes. Thus, strong rationale exists supporting the need for comprehensive examinations of the xMHC not limited only to the classical HLA loci, which historically have been given the most attention in disease association studies.

Because childhood ALL is suspected to be caused by an adverse immune response, possibly triggered by infectious exposures, and because the xMHC includes a wide range of genes with potential relevance to leukemogenesis, the xMHC is a highly appropriate candidate region for investigation. Indeed, there is currently a substantial collection of studies that have evaluated a potential involvement of xMHC loci in childhood ALL risk. In this review, we aimed to provide the reader with a synthesis of the current epidemiological evidence available to date regarding the association between genetic variation of the xMHC and risk of childhood ALL.

## Classical HLA Genes

Human leukocyte antigen is polygenic and encodes heterodimeric cell surface glycoproteins involved in antigen presentation that selectively interact, in a peptide-binding groove, with short peptide fragments derived from non-self and self-proteins. The classical HLA class I molecules (HLA-A, HLA-B, and HLA-C) present foreign antigens derived predominantly from intracellular infection, and initiate CD8^+^ T-cell responses, while class II molecules (HLA-DRB1, HLA-DRB3, HLA-DRB4, HLA-DRB5, HLA-DQB1, HLA-DQA1, HLA-DPB1, and HLA-DPA1) present antigens derived from the extracellular space to CD4^+^ T helper cells, which, in turn, activate macrophage and B-cells. The genes encoding HLA molecules are highly polymorphic with up to many hundreds of alleles segregating at a single locus (16). Because most are confined to the region encoding the peptide-binding groove, the HLA polymorphisms have functional relevance and significance for disease susceptibility. Sequence homology and epitope sharing between alleles and loci in the HLA genes is evident from an inspection of the sequences (17) for current alignments^1^. Selective pressures for balanced polymorphism act to increase amino-acid diversity of HLA molecules in populations, but stoichiometric constraints influence the amino-acid make-up of an HLA molecule, and both influence the types of peptides that can be bound by the HLA molecule (18).

Scrutiny of the HLA system in leukemia susceptibility in humans began in the 1960s following landmark studies by Lilly and colleagues who demonstrated that leukemogenesis by the Gross leukemia virus in mice is strongly influenced by H-2 (the MHC system in mice) type (19). Utilizing the best available typing methods and knowledge of the HLA system at the time, the earliest studies in humans showed a link with class I loci such as HLA-A2 antigen serotype (20–22), but were limited by relatively small study populations and absence of restriction to age groups (i.e., children or adults) and sub-types. In addition, these studies used low-resolution HLA typing methods based on serological recognition of cell surface antigens, which do not fully capture the allelic diversity of HLA. Over time, with advances in understanding of HLA genetics, development of higher resolution molecular typing techniques, and availability of well-defined study populations, more refined and methodologically rigorous evaluations of HLA in childhood leukemia susceptibility has been possible. Here, we summarize the results of 23 previous studies, the first of which was published in 1980, that specifically examined leukemia in children in relation to genetic variation at the classical class I and class II HLA loci (Table 1).

**Table 1:** **Studies evaluating the association between HLA genetic variation and ALL risk specifically in children**.

Reference	Region	Study population	Locus	Main results^a^	Comments
von Fliedner et al. (35)	Switzerland	31 ALL; 123 controls	HLA-DR (antigen)	DRw7 over-represented in childhood ALL compared to controls	Adult ALL and AML results also available in report
				Evidence of increased DRw7 homozygosity in patients vs. controls	
Davey et al. (25)	USA	94 ALL; 376 controls	HLA-A (antigen) HLA-B (antigen)	HLA-A and HLA-B antigens: no difference in distribution between groups	HLA-A9 associated with first remission duration and survival
von Fliedner et al. (26)^b^	Switzerland	55 Families with child affected with ALL	HLA-A (antigen)	HLA sharing among parents of leukemic children was increased for HLA-B (*p* = 0.023) and HLA-DR antigens (*p* = 0.003) compared to expected	Mating of certain shared alleles in HLA is associated with risk in offspring
			HLA-B (antigen)	
			HLA-C (antigen)	
			HLA-DR (antigen)	
Muller et al. (27)	Germany	142 ALL (75, aged <11 years)	HLA-A (antigen) HLA-B (antigen) HLA-C (antigen) HLA-DR (antigen)	HLA-Cw7: higher frequency in ALL compared to both control groups (corrected *p* = 0.00064)	*p*-Value corrected for multiple comparisons (73 tests)
		280 Local controls		
		1,053 Caucasian controls		Difference was strong in ALL age ≥11 years compared to controls; in children (age <11), not significant after correction	
Cameron et al. (28)	Trinidad, West Indies	10 ALL; controls	HLA-A (antigen) HLA-B (antigen) HLA-C (antigen)	HLA-B40: higher frequency in ALL compared to controls (*p* < 0.05)	
				HLA-B5: no carriers in cases, but 37.8% in controls (*p* < 0.05)	
Taylor et al. (42)	United Kingdom	63 BCP-ALL; 92 adult and 82 infant controls	HLA-DPB1 (4-digit allele)	DPB1*02:01: RR = 2.9, *p* < 0.05	
				Patients are three to four times more likely to be heterozygous for DPB1*02:01/*03:01,/*04:01,/*04:02	
Dearden et al. (48)	United Kingdom	62 BCP-ALL; 76 newborn controls	HLA-DQB1 (4-digit allele)	DQB1*05: RR = 2.54, *p* = 0.038 DQB1*0501: RR = 2.18, *p* = 0.095 DQB1*0501 more common in males DQB1*0601 more common in females	DQB1*0501 and DPB1*0201 ALL association appeared independent
					Amino-acid motifs evaluated
Taylor et al. (49)	United Kingdom	62 BCP-ALL; 78 newborn controls	4-Digit allele	Male-specific associations:	Association with specific HLA-DQA1 and HLA-DQB1 amino-acid motifs evaluated
			HLA-DQA1 HLA-DQB1	DQA1*0101/*0104: OR = 4.06 (1.42–10.17)	
				DQA1*0201: OR = 0.40 (0.18–0.96)	
				DQA1*03: OR = 2.62 (1.07–6.02)	
				DQA1*0101 and DQB1*0501 co-occurrence: OR = 3.73 (1.19–10.3)	
Ghodsi et al. (32)	United Kingdom	94 BCP-ALL; 136 infant controls	HLA-C (typed for NK1 or NK2 ligand)	NK1 and NK2 frequencies showed no statistically significant difference between ALL and controls	Excess of NK2 homozygous patients compared to controls, particularly in females
Dorak et al. (37)	United Kingdom	114 ALL; 325 newborn controls	2-Digit allelic type: HLA-DRB1 HLA-DRB4 (DR53) HLA-DRB3 (DR52) HLA-DRB5 (DR51)	Male-specific associations DRB1*0401 (DR4): OR = 2.9 (1.6–5.4) DRB4*01 (DR53): homozygosity, OR = 11.7 (4.9–28.0) *DRB3* (DR52): OR = 0.4 (0.2–0.7)	HLA-DRB1*04 is part of DR53 supertype, thus, not independent Recessive nature of HLA influences on childhood ALL
Taylor et al. (43–45)	United Kingdom	529–687 BCP-ALL depending on study; 864 newborn controls, 409 childhood solid tumors	HLA-DPB1 (4-digit allele and supertype)	DPB1*0201: OR = 1.83 (1.34–2.41) Other *DPB1* alleles encoding peptide motifs present in DPB1*02:01 associated DPB1*01:01 associated with *TEL-AML1*+ and hyperdiploid ALL *DP2* supertype: OR = 1.7 (1.3–2.1) *DP8* supertype: OR = 3.2 (1.5–7.0) *DP1* supertype: OR = 0.5 (0.4–0.7) DPB1*0601: OR = 3.6 (1.5–8.6) and associated with non-BCP-ALL sub-types	Results indicated in table are for BCP-ALL Other ALL sub-types (pro-B ALL, T-ALL, unclassifiable) also evaluated Amino-acid motifs evaluated Multiple comparisons considered in all analyses
Dorak et al. (38)	Turkey	114 ALL; 118 controls	2-Digit allelic type:	Male-specific associations	Adult ALL results also available in report
			HLA-DRB1	DRB1*04: OR = 3.08 (1.14–6.53)	
			HLA-DRB4 (DR53)	DRB1*13: OR = 0.32 (0.14–0.74)	
			HLA-DRB3 (DR52)	*DRB4* (DR53): OR = 2.85 (1.42–5.70)	
			HLA-DRB5 (DR51)	*DRB3* (DR52): homozygosity, OR = 0.37 (0.15–0.89)	
Yari et al. (39)	Iran	22 ALL; 466 controls	HLA-DRB1 (2-digit allele)	DRB1*13: lower frequency in ALL compared to controls (*p* = 0.044)	Adult ALL results also available in report
					Moderate case-control difference in frequency for HLA-DRB1*04, not significant
Wang et al. (40)	China	162 ALL; 1,000 cord blood controls	HLA-DRB1*15	DRB1*15: RR = 1.51, *p* = 0.018	
			HLA-DRB5	
Morrison et al. (33)	United Kingdom Mexico	UK: 114 ALL; 388 newborn controls Mexico: 100 ALL; 253 adult controls	HLA-DRA (rs3135388, DRB1*1501 proxy) HLA-C (rs9264942, Cw5 proxy) Class III loci SKIV2L (rs419788) TNXB (rs3130342) Non-MHC locus	Female-specific associations *HLA-DRA* (rs3135388): OR = 2.6 (1.5–4.5) *HLA-C* (rs9264942): OR = 0.4 (0.2–0.7) *SKIV2L* (rs419788): OR = 1.8 (1.1–2.9) *TNXB* (rs3130342): OR = 2.2 (1.2–4.3) Male-specific association *IFNG* (rs2069727): OR = 0.6 (0.4–1.0)	Focused on loci associated with multiple sclerosis and systemic lupus erythematosus Associations for SKIV2L and TNXB were attenuated after adjusting for HLA-DRA
			IFNG (rs2069727)		
Ozdilli et al. (29)	Turkey	100 ALL; 90 AML; 100 controls	HLA-A (antigen) HLA-B (antigen) HLA-DRB1 (allele, 2-digit)	HLA-A23: RR = 0.21 (0.04–1.03) HLA-B7: RR = 0.35 (0.13–0.96) HLA-DRB1*04: RR = 2.11 (1.13–3.96)	Gender-specific ALL associations suggested AML associated with HLA-A11, HLA-B38, -B49, and -DRB1*15
Hosking et al. (23)	United Kingdom	824 BCP-ALL; 4,737 adult controls	4-Digit allelic types: HLA-A	No statistically significant associations after correction for multiple testing Six alleles associated at an uncorrected *p* < 0.05: HLA-A*26, HLA-A*29, HLA-C*02, HLA-C*08, DQB1*02, DQB1*03	HLA alleles imputed using a reference database of SNP haplotypes carrying known HLA alleles
			HLA-B	
			HLA-C	
			HLA-DQA1	
			HLA-DQB1	
			HLA-DRB1	
Klitz et al. (24)	USA	2,438 medically refractory ALL; 41,750 adult donor controls	2-Digit allelic types:	Continuum of risk associations observed for *HLA-A, HLA-B*, and *HLA-DRB1* loci (see Figure 2)	
			HLA-A	
			HLA-B	
			HLA-DRB1	
Orouji et al. (50)	Iran	107 ALL; 110 controls	HLA-DQB1 (allele)	DQ2 (*02:01): RR = 0.75, *p* = 0.049	DQ5 also found associated with adult ALL in this study
				DQ5 (*05:01–*05:04): RR = 1.89, *p* = 0.001	
				DQ7 (*03:01, *03:04): RR = 1.48, *p* = 0.003	
Urayama et al. (47)	USA	585 ALL; 848 controls	4-Digit allele types:	DPB1*01:01: OR = 1.43 (1.01–2.04)	Interaction observed between *DP1* supertype and 2 proxy for immune modulation
			HLA-DPA1	*DP1* supertype in hyperdiploid ALL: OR = 1.83 (1.20–2.78)	
			HLA-DPB1	

*^a^ HLA nomenclature continues to evolve as information about its extraordinary genetic variation becomes available. The nomenclature in this table mostly reflects that which was current at the time and used in the manuscripts cited*.

*^b^ With the exception of this one family-based study, all others in the table utilized a case-control study design*.

### HLA class I loci

#### HLA-A and HLA-B

With the exception of two reports (23, 24), studies of *HLA-A, HLA-B*, and *HLA-C* associations with childhood ALL risk have been based on serotypes rather than allelic level classifications. The HLA-A2 antigen association found in the previous generation of studies of ALL (all age groups) was not observed in subsequent studies conducted specifically in children. In one of the earliest studies of HLA and childhood ALL risk, Davey et al. (25) observed no difference in distribution of HLA-A and HLA-B antigens between patients and controls. Among four additional studies that investigated HLA-A antigens in childhood ALL (26–29), only one reported an association with this locus, but with the HLA-A23 serotype (29). An association with the *HLA-B* locus was found in three of these studies (26, 28, 29), one of which was a family-based study comprising 55 families with a child affected with ALL (26). A comparison made between observed vs. expected numbers of shared antigens among parents with ALL offspring showed increased compatibility for HLA-B antigens, suggesting a role for this locus as a recessive determinant. Using the case-control design, one study reported higher HLA-B40 and lower HLA-B5 antigen frequencies in Trinidadian cases compared to controls (28), and another study conducted in Turkey reported a reduced risk associated with the HLA-B7 antigen (29).

**Figure 2:**
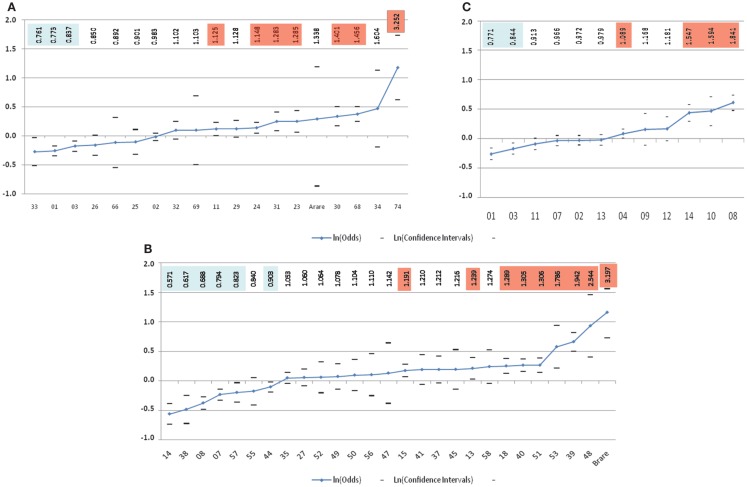
**Risk of medically refractory childhood ALL according to 2-digit HLA alleles at *HLA-A* (A), *HLA-B* (B), and *HLA-DRB1*(C) (24)**. The vertical scale is in ln(Odds) with odds values listed alone the top highlighted in blue for favorable and orange for predisposing-allele.

The lack of consistent results for HLA class I loci from these mostly small studies (all less than 100 patients) utilizing low-resolution HLA typing complicates interpretation. Addressing these shortcomings, in the largest study to date evaluating HLA and childhood ALL risk, Klitz et al. (24) utilized HLA allelic data from the National Marrow Donor Program (NMDP) in an analysis of 2,438 medically refractory childhood ALL patients being considered for or undergoing hematopoietic stem cell transplantation and 41,750 controls that were matched on age (2–15 years), sex, and geographical location in the United States. This comprehensive analysis of the *HLA-A* and *HLA-B* loci revealed a spectrum of HLA associations conferring predisposition to or protection from refractory disease (Figure 2). Four significantly (*p*-value <0.01) protective *HLA-A* alleles (A*33, A*01, A*03, and A*26) and six predisposing-alleles (A*24, A*31, A*23, A*30, A*68, and A*74) were found, with 50% of alleles as a whole either predisposing or protective in this high-risk ALL population (Figure 2A). *HLA-B* allelic associations revealed six protective alleles (B*14, B*38, B*08, B*07, B*57, and B*44) and eight predisposing (B*15, B*18, B*40, B*51, B*53, B*39, B*48, and a binned category “rare” with <50 copies) (Figure 2B). As with *HLA-A*, they showed a continuum of odds ratios ranging from 0.57 to 3.20, with more predisposing than protective alleles, and 24.0% of alleles being neutral with respect to disease. These analyses show a spectrum of HLA associations and indicate that studies with larger sample sizes might clarify the predisposing, neutral, or protective status of alleles whose confidence intervals are close to, or include, an odds ratio of 1.0. Further, each allele may be regarded as a marker effect for sequences in LD with the antigen.

Next, the authors classified genotypic groups according to allelic composition, with alleles being protective, predisposing, or neutral. Single allele effects for protective-neutral heterozygotes and homozygous protective genotypes both conferred significantly greater protection. In contrast, homozygous were significantly more predisposing than heterozygous predisposing genotypes. Haplotypes were similarly grouped, with homozygous predisposing-allele haplotypes more disposed to medically refractory childhood ALL than heterozygous predisposing-allele haplotypes. Haplotypes bearing favorable alleles revealed a different pattern, with significant protection only from *HLA-B* haplotypes with protective alleles, while the mixed predisposing/protective haplotypes were neutral, suggesting that these cancel each other out. Overall, these results indicate novel qualities of HLA loci in medically refractory childhood ALL. In these analyses, *HLA-A* appeared to have the weakest effect on ALL predisposition and protection in these children. *HLA-B* alleles show substantial protective and predisposing effects, with stronger effects seen in homozygous states. This continuum in disease susceptibility suggests a system in which many alleles take part in disease predisposition based on differences in binding affinity to one or a few peptides of exogenous origin, adding to the numerous studies pointing to a role for an infectious origin in childhood ALL.

#### HLA-C

Childhood ALL associations with *HLA-C* have also been reported, but like the other class I loci, the associated antigens have not been consistent across studies. Muller et al. (27) in a German study compared ALL patients (all ages) to two different control groups and found statistically significant associations with HLA-Cw7 even after correction for multiple comparisons. However, the association was less evident when restricted to patients less than aged 11 years. Associations with HLA-Cw3 and HLA-Cw4 have also been noted in studies of ALL conducted among children and adults (30, 31). Since different HLA types of a specific locus may encode proteins with overlapping functional specificities, a situation of having multiple ALL-associated HLA types is a possibility. Ghodsi et al. (32) examined this by distinguishing *HLA-Cw* types with respect to whether they encode either NK1 or NK2, ligands that interact with receptors on natural killer cells. The results did not support a role for the *HLA-C* locus in childhood ALL susceptibility. Building on previous observations of an increased risk of ALL in children of women with autoimmune disorders, Morrison et al. (33) specifically examined multiple sclerosis and systemic lupus erythematosus risk markers. A childhood ALL association with the single nucleotide polymorphism (SNP) rs9264942, a marker for HLA-Cw5, was found specifically among females similar to the protective effect observed in multiple sclerosis.

### HLA class II loci

#### HLA-DRB

Suggestion of a putative role of the HLA class II DR antigen on ALL risk was first reported in the late 1970s (34) and showed an association with DR7. Additional evidence specifically in childhood ALL was available shortly after based on studies by Von Fliedner et al., one of which confirmed the DR7 association (35) and the other showing a higher than expected sharing of DR antigens among parents of children with leukemia (26).

Subsequent inspections at the allelic and supertype level of *HLA-DRB1*, together with the expressed DR beta chain paralogs (*HLA-DRB3, -DRB4*, and *-DRB5*) have yielded some of the most consistent evidence to date of an HLA link with childhood ALL risk. Dorak et al. (36) reported a strong male-specific childhood ALL association with homozygosity for the HLA DR53 supertypic antigen (encoded by *HLA-DRB4*), consistent with effects seen in other major forms of leukemia such as chronic lymphocytic and myeloid leukemia and acute myeloid leukemia (36). A few years later, using an expanded series of cases and controls, these findings were confirmed in a comprehensive examination of all DR beta chain genes (37). An effect specifically in males was found for DRB1*04, a common *DRB1* allelic group, and part of the DRB4 haplotype expressing the DR53 antigen. Examination of *DRB3, DRB4*, and *DRB5* (encoding the DR52, DR53, and DR51 supertypes, respectively) showed considerably higher DRB4*01 (DR53) homozygosity rates in patients compared to controls that was specific to males. In addition, a decreased *DRB3* frequency was observed in patients compared to controls, again in males only. These findings were subsequently replicated in an independent series of high-risk and relapse childhood ALL patients and controls and additionally noted marked protective effects for DRB1*12 and DRB1*13, alleles linked to the *DRB3* (DR52) haplotypic group (38).

Additional evidence for a *DRB1* association comes from three other studies. Two of these, an Iranian (39) and a Turkish (29) study, both showed protective and predisposing effects of DRB1*13 and DRB1*04, respectively. The third was the large NMDP study of European Americans described earlier (24). Similar to their findings for the class I *HLA-A* and *HLA-B* loci, Klitz et al. described a spectrum of associations at *DRB1* with odds ratios ranging from 0.77 to 1.84, and observed an imbalance toward predisposing effects including DRB1*04, DRB1*14, DRB1*10, and DRB1*08 (Figure 2C).

Finally, DRB1*15, a risk allele for multiple sclerosis, has also been implicated in the susceptibility to childhood ALL. At least two recent studies have reported an association, one in a Chinese population comprising 162 childhood ALL cases and 1,000 controls (40), and another in a UK population using the SNP rs3135388 as a proxy for DRB1*15:01 (33). The latter study examined the effects by gender and observed the association only in females, which is consistent with a female-specific effect of the allele also seen in multiple sclerosis (41).

#### HLA-DP

The first study to identify an association of *HLA-DPB1* types with childhood ALL was a preliminary study by Taylor et al. (42), based on comparison of typing data from childhood common ALL cases and two sets of controls (42). The study found that the allele DPB1*02:01 was more than twice as common among children with ALL as in either set of controls. These findings were confirmed and extended in 2002 (43) in the United Kingdom Childhood Cancer Study (UKCCS) demonstrating that DPB1*02:01 was significantly more common among a large series of 875 childhood ALL patients than in either of two control groups (corrected *p*-value <0.05). A negative association of DPB1*01:01 with childhood ALL was also noted. Further, additional analyses generated a global *p*-value <10^−6^ for a difference between *HLA-DPB1* phenotype frequencies between common ALL and controls and indicated that alleles other than DPB1*02:01 also contribute to susceptibility. The authors examined the distribution of key peptide-binding residues in the pocket domains of these alleles, which indicated that there was increased sharing of these between HLA molecules predicted to contribute to disease risk, compared to those encoded by other alleles. Examination of the phenotype frequencies of specific peptide-binding pocket residues demonstrated that significant associations with childhood ALL susceptibility were almost exclusively restricted to those peptide motifs present in DPB1*02:01; with significantly different associations for total leukemias, total ALL, common ALL, *TEL-AML1*+ ALL, and high-hyperdiploid ALL, compared to controls.

In an extension of this work, Taylor et al. (44) defined six *HLA-DPB1* supertypes, based on amino-acid dimorphisms at positions 11 (G/L), 69 (E/K), and 84 (G/D) (Table 2), consisting of unique peptide haplotypes encoded by the hypervariable second exon of *HLA-DPB1*, which group alleles predicted to demonstrate functional similarities with regard to antigen binding. Among the UK childhood leukemia cases and controls, of the six supertypes examined, three (*DP2, DP6*, and *DP8*) showed evidence of increased frequencies in cases, although this was only significant in *DP2* and *DP8* after correction for multiple testing. These two supertypes were also significantly increased in BCP-ALL after correction, whereas *DP6* appeared to be more common among non-BCP leukemia cases (*p*-value = 0.007). Moreover, the *DP1* supertype frequency was much lower among BCP-ALL cases (6.4%) compared to controls (11.1%; *p*-value <10^−5^), a finding which did not extend to non-BCP leukemia. Stratification of cases by age at diagnosis revealed a significant association of the *DP2* supertype with cases occurring at the typical peak age for diagnosis for BCP-ALL (3–6 years; *p*-value = 10^−4^), but not among cases diagnosed before 3 years or after 6 years of age. By contrast, the decreased frequency of the *DP1* supertype was apparent in all groups of age at diagnosis. In a separate analysis of supertype distributions in ALL sub-types (44), a paucity of another *DP1* supertype (GKD) was found among cases with *TEL-AML1*^+^ and high-hyperdiploid karyotypes relative to controls.

**Table 2:** **HLA class II *DPB1* supertype distributions (percentage of individuals carrying) reported in the UKCCS and NCCLS studies**.

DPB1 supertype^a^	NCCLS%		UKCCS%	
	Cases	Controls	Cases	Controls^b^
DP2 (GEG)	24.8	24.5	18.3	13.3
DP4 (GKG)	79.2	80.9	82.9	72.4
DP6 (LED)	14.2	15.0	11.6	12.9
DP3 (LKD)	19.5	20.2	20.9	20.9
DP8 (GED)	3.4	2.2	2.4	0.8
DP1 (GKD)	16.2	12.7	13.5	17.7

*UKCCS, United Kingdom Childhood Cancer Study; NCCLS, Northern California Childhood Leukemia Study*.

*^a^ Supertypes based on amino-acid dimorphisms at positions 11 (G/L), 69 (E/K), and 84 (G/D) (44)*.

*^b^ Infant controls*.

Further investigation of the association of the *DP6* supertype with leukemia (45) found that, of the seven alleles included in this supertype, only DPB1*06:01 was significantly associated with childhood leukemia (corrected *p*-value = 0.02), including total ALL, AML, BCP-ALL, Pro-B ALL, and T-ALL. Further analyses indicated that, of all *DPB1* alleles and supertypes, DPB1*06:01 was the most strongly associated with leukemia. The genotype DPB1*04:01/*06:01 is particularly strongly associated with ALL (OR, 95% CI; 10.6, 2.4–47.4). Transmission disequilibrium tests indicated that this allele was over-transmitted to children who go on to develop leukemia (76.9%; *p*-value = 0.03). Subsequent extended analysis of *DPB1* allele transmission revealed no evidence of over- or under-transmission of *DP1* or *DP2* supertypes among UK leukemia cases, although there was a suggestion of under-transmission of other rare alleles (46).

More recently, Urayama et al. (47) investigated HLA-DP in relation to childhood leukemia in the Northern California Childhood Leukemia Study (NCCLS), which includes an ethnically heterogeneous sample consisting of two main populations; non-Hispanic white and Hispanic. This study found no gross differences in *HLA-DPA1* allele frequencies or *DPA1-DPB1* haplotypes between cases and controls. However, analyses conditioned on age, sex, and ethnicity identified a statistically significant association with DPB1*01:01. Using the supertype classification of Taylor et al. (44), this translated as an association of *DP1*, with increased ORs in both non-Hispanic whites and Hispanics, significant for high-hyperdiploid ALL (*p*-value = 0.005). In contrast, Taylor et al. found the *DP1* supertype was associated with decreased risk. Importantly, as an extension beyond previous studies of *HLA-DPB1* and childhood leukemia, the California study also examined interactions between proxies for delayed infections in childhood (daycare attendance, ear infections, and breastfeeding) with the observed *DP1* association. Interactions were detected between DP1 and two proxies for early immune modulation, older sibling (*p*-value = 0.036), and breastfeeding (*p*-value = 0.094) in the risk of childhood ALL. Although the power to detect interactions between *DP1* and daycare/ear infections in the first year was limited, the point estimates were in the expected direction should the association be related to delayed infection in childhood ALL, as hypothesized by Greaves (4).

The observation of opposing effects of the *DP1* supertype on childhood ALL susceptibility in two apparently ethnically similar populations is intriguing, especially as both studies narrowed the significant associations to the high-hyperdiploid subtype of ALL. Comparison of supertype frequencies suggests heterogeneity between the two populations (Table 2). The *DP1* supertype appears to be less common in the NCCLS non-Hispanic white controls (12.7%) compared with those employed in the UKCCS (17.7%). Conversely, the frequency of *DP2* is lower among UKCCS controls (13.3%) compared to the NCCLS (24.5%) (Table 2). These underlying differences may indicate differences in selective pressure acting on the two geographically distinct populations, or alternatively be a consequence of other processes, such as genetic drift.

#### HLA-DQ

In comparison to the other class II loci, there are relatively few studies examining HLA-DQ in relation to childhood ALL risk. Two of the three previous studies were based on the same study population with an initial report by Dearden et al. (48) describing for the first time an ALL association with the DQB1*05:01 allele that was independent of their previously identified DPB1*02:01 effect. In their subsequent report, Taylor et al. (49) conducted a more detailed examination of HLA-DQ that included the tightly linked *HLA-DQA1* locus. Their findings showed an increased risk associated with DQA1*0101/*0104 exclusively in males and a protective effect associated with HLA-DQA1*0201. A joint analysis of *DQA1* and *DQB1* indicated a strong effect associated with carriers of both DQA1*0101/*0104 and DQB1*0501 and was seen only in males. Interestingly, in a third *DQB1* study conducted in Iranian childhood ALL patients and controls by Orouji et al. (50), a consistent predisposing effect was seen for DQB1*05 (DQ5), and additionally a protective and predisposing effect for DQB1*02:01 (DQ2) and DQ7 (DQB1*03:01, *03:04), respectively.

## Candidate Gene Studies of Non-HLA Loci in xMHC

The vast majority of genes in the xMHC that have been studied with respect to childhood leukemia are HLA; however, a number of non-HLA genes in the xMHC region have also been examined. While in general these genes have not been studied in multiple populations, they highlight the potential importance of genetic variation in the xMHC in the etiology of childhood leukemia.

Han et al. (51) conducted a case-control study of the role of immune response genes in total childhood leukemia in Korea. Among the studied loci were two genes in or near the xMHC region, *TNF* and *NFKBIE* (6p21.1). Using gene-wise analytical methods to adjust for examination of multiple SNPs in each gene, the authors observed no significant associations for any of the genes included. This study included a relatively small sample size (63 cases and 148 controls) and examined total childhood leukemia, including ALL (59%) and AML (26%). Results for individual leukemia sub-types were not presented.

Similarly, in a study of xenobiotic transport and metabolism genes, Chokkalingam et al. (52) examined whether *GPX6/GPXP3* (6p22.1) and *TPMT* (6p22.3) were associated with childhood ALL risk among 377 cases and 448 controls. The authors found no significant associations for the studied SNPs in these genes either individually or in haplotype analyses.

In a study of genes involved in p53 signaling, Do et al. (53) examined a number of loci within the xMHC region. Among 114 ALL cases and 414 controls, they found significant associations with SNPs in *BAT3/BAG6*, a molecular chaperone involved in transcriptional control of the p53 tumor suppressor. However, no associations were observed for other genes within the xMHC, including *DAXX, LTA, DDR1*, and *IER3*.

The human hemochromatosis gene (*HFE*) modulates serum iron levels, which have been linked to risk of several cancers. Interestingly, evidence linking risk of childhood ALL to high birthweight has been reasonably consistent (54), and it is possible that this observation may reflect or be modulated by serum iron levels. In a study of 163 ALL cases and 995 controls, Dorak et al. (55) explored this hypothesis and found that just one of 15 SNPs in *HFE* was significantly associated with childhood ALL risk. This SNP, rs9366637, in intron 1 of *HFE*, was also associated with birthweight. However, among the 14 other studied SNPs were two well-known functional SNPs, rs1800562, and rs1799945, which were not associated with risk of childhood ALL.

Heat shock proteins are molecular chaperones that may play a role in both adaptive and innate immune responses to tumors and virus-infected cells. Ucisik-Akkaya et al. (56) examined functional polymorphisms in three HSP genes that reside in the class III region: *HSPA1L, HSPA1A*, and *HSPA1B*. Among 114 ALL cases and 414 controls from the UK, they found that rs1061581, in *HSPA1B*, was significantly associated with childhood ALL risk. The association was replicated in a separate set of 100 ALL cases and 253 controls from Mexico (56). The authors noted that although this SNP is in LD with *HLA-DRB3*, which was associated with childhood ALL in the UK dataset, the *HSPA1B* association was independent of *HLA-DRB3*. SNPs in *HSPA1L* and *HSPA1A* showed no significant associations with childhood ALL risk. Using the same UK and Mexican populations, Morrison et al. (33) examined the role of multiple sclerosis susceptibility factors in childhood ALL risk. Included were two non-HLA genes in the xMHC region: *SKIV2L* and *TNXB*, which were not found to have significant single SNP associations with childhood ALL risk.

In summary, reports of childhood ALL risk associated with non-HLA loci within the xMHC provide limited evidence that the MHC may have relevance to childhood leukemia etiology above and beyond the HLA. The reports described above include a somewhat arbitrary and incomplete representation of the non-HLA genes in the xMHC. Furthermore, with the exception of the Morrison et al. and Ucisik-Akkaya et al. reports, which included independent replication populations, none of the genes described in this section were examined in more than one study population, and none were described in more than one report.

## xMHC Association Mapping Studies

Hosking et al. (23) used data generated in the UKCCS genome-wide association study (GWAS) to conduct an analysis of BCP-ALL association across a 4.5-Mb xMHC region. They performed single SNP comparisons, alongside a sliding window haplotype analysis and imputation of HLA genotypes at the major class I (*HLA-A, -B*, and *-C*) and class II (*HLA-DQA1, -DQB1*, and -*DRB1*) loci, with data derived from UK population samples as controls (824 cases and 4,737 controls). No associations, significant after correction for multiple testing were observed; however, the strongest single SNP-based association was with rs3135034 (*p*-value = 0.0017; not significant after adjustment for multiple testing), which is approximately 92 kb telomeric of *HLA-DPB1*, between the *BRD2* and *HLA-DOA* loci. In addition, after correction for multiple testing, no significant associations were observed with imputed HLA alleles. The strongest (non-significant) associations were found for A*26:01, A*29:02, B*14:02, C*02:02, and C*08:02 class I alleles and DQB1*02:01, DQB1*03:02, DQB1*03:03, and DRB1*13:02 class II alleles.

Recently a high-density SNP analysis of the MHC region in the NCCLS was conducted (57). In single SNP analyses this study identified one locus, rs9296068, as significantly associated with BCP-ALL risk after correction for multiple testing (OR = 1.40, 95% CI = 1.19–1.66, corrected *p*-value = 0.036). Of interest in the context of the UKCCS MHC study (23), this SNP lies only approximately 37 kb from rs3135034, the most strongly associated UK xMHC SNP, upstream of the HLA-DOA transcription start site. Sliding window haplotype analysis was also undertaken as part of this study, yielding two significantly associated MHC haplotypes, the first close to the *TRIM27* gene in the extended class I region (rs1237485-rs3118361-rs2032502-rs7747023; nominal global *p*-value = 2.7 × 10^−5^) and the second including rs9296068 and flanking *HLA-DOA* (rs423639-rs7754316-rs9296068; nominal global *p*-value = 9.2 × 10^−5^). The haplotype showed little evidence of correlation with the *DP1* supertype, previously found to be associated with ALL susceptibility in the same group of patients, and analysis adjusting for carriers of the *DP1* supertype indicated an independent effect for rs9296068 (*p*-value = 3.0 × 10^−4^). In contrast to the Hosking et al. study, this investigation did not extend to the imputation of additional SNPs in the MHC region or the prediction of classical HLA types. This fact may have contributed to the identification of significant associations not apparent in the UK data; these may have been missed in the UK study as a result of the additional burden of correction for multiple testing.

Although the two SNPs identified in the UK and California BCP-ALL MHC association studies were physically close to one another, neither SNP showed any indication of association in the reciprocal studies. However, the two SNPs flank a strong meiotic recombination hotspot, known as *DNA3* (58). It is established that commonly used methods of genetic association mapping would not be expected to identify associations under recombination hotspots, since they rely on positive LD, which hotspots lack by definition. Hence, it is possible that these two SNPs tag a *HLA-DOA* association in both populations, partially masked by the strong *DNA3* hotspot.

This hypothesis led to another study, consisting of a detailed investigation of haplotypes and recombination rates in this region of the MHC in UK childhood ALL cases and controls (Thompson et al., submitted). Using the program PHASE (59) to predict haplotypes and recombination rates among UKCCS ALL patients and UK population controls from data generated in the UKCCS GWAS (60), this study found evidence for an association with recombination rates at *DNA3* and childhood ALL. Moreover, a novel analysis using LDsplit (61) showed that loci on both sides of the hotspot, including rs9296068 and rs3135034, contribute to recombination at *DNA3*. Using data from an Icelandic study (62) the authors demonstrated that recombination at *DNA3* is strongly affected by *PRDM9* genotype, the relevance of which is underlined by a recent publication demonstrating a marked increase in the prevalence of rare *PRDM9* alleles among parents of childhood B-cell ALL patients, and altered recombination patterns in patients, in two North American populations (63).

Evidence from these analyses indicates that there are two physically close, but weakly linked risk loci for childhood ALL at *HLA-DOA* and *HLA-DPB1* in the MHC class II region, both substantiated by results from two separate studies in the UK and California. However, data from these studies are inconsistent in terms of the nature of the *HLA-DPB1* allelic associations with risk of ALL and in the precise location of the association in the vicinity of *HLA-DOA*. The former is complicated by apparent underlying population differences in frequencies of HLA-DP, but may be attributable to geographic variations in exposures to antigens such as infectious agents. The weak but significant associations in both studies with loci flanking the *DNA3* meiotic recombination hotspot are suggestive of an association with *HLA-DOA*, a molecule critical for HLA class II antigen processing, masked by the breakdown of LD in this region. The increased rates of recombination at *DNA3* may further exacerbate the heterogeneity of genotypes at this locus. Previously identified functionally important variants within recombination hotspots have been associated with recombination mediated sequence alterations (64, 65); it remains to be determined whether this applies to *HLA-DOA* in the context of childhood ALL.

## Conclusion and Future Directions

Results based on mostly low-resolution genotypes and HLA technology that has evolved over time, compounded by small study sizes and conducted independently across populations of different ancestral backgrounds, present challenges to fully interpreting the collective HLA evidence. Thus far, there appear to be no striking childhood ALL genetic susceptibility loci in the xMHC that are similar in nature and magnitude to those seen for autoimmune and infectious diseases. However, mounting evidence does suggest that more modestly associated susceptibility loci showing population and subtype specificity may exist. In particular, this is exemplified by multiple previous studies showing male-specific effects for DR53 and evidence of *HLA-DPB1* BCP-ALL associations with potentially more detectable effects when in the presence of certain environmental exposures. A role for *HLA-DPB1* or a gene localized to that region is also supported by two independent mapping studies of the xMHC demonstrating a need for targeted fine-mapping efforts. Further, a highly powered study of children with medically refractory ALL, those being considered for or going to stem cell transplantation, has revealed highly significant associations to both HLA class I (*HLA-A* and -*B*) and class II (*HLA-DRB1*), results which may shed light on the role of HLA in the less severe disease form of pediatric ALL. While this is a noteworthy study due to its methodological rigor that is unique over previous studies, validation in comparable and appropriately large study populations is still required.

There are several opportunities for improvement in future studies given our currently expanded knowledge of the genetic complexities of the xMHC (14, 66, 67), technological advances in genomic characterization and high-resolution HLA genotyping (68), and increased recognition of the importance of international collaboration and independent validation (69). The genomic complexities of the xMHC, such as long-range LD that gives rise to extended haplotypes and extensive genetic variation of the region, are now well-characterized and facilitate the initial localization of genomic regions, but complicate the effort to fine-map causal genetic loci to disease risk (70). The classical HLA genes are the most widely studied of the xMHC loci and have been associated with dozens of diseases, but in many cases, including childhood ALL, it is not clear whether the associations can be unambiguously linked to the HLA gene or instead indicate an association with an adjacent causal locus in LD. To address this, comprehensive examination of genetic variability of the entire xMHC, including high-resolution HLA allelic typing, is needed and is currently achievable using recently developed technologies. High-density MHC SNP genotyping studies, two of which were described in this review (23, 57), are a step in this direction, but they lacked direct allelic typing of all classical HLA loci. The UKCCS applied previously derived methods (71, 72) to impute HLA alleles based on GWAS-derived SNPs for six of the nine classical HLA loci. While this is a reasonable alternative to a costly and intensive HLA allelic typing effort of all loci, whether such imputation methods can replace classical typing is still questionable and should be considered on an individual study basis depending on sample size, ancestral composition of the study population, and availability of SNP and HLA data from a suitable reference panel (73).

Methods for routine next-generation DNA sequencing-based HLA typing have recently been developed (68) and provide the highest quality and resolution of allelic types without the need for time-consuming efforts to resolve allelic ambiguities in heterozygotes. Full implementation of these methods has recently been applied to a case-control study of follicular lymphoma, demonstrating their feasibility for use in disease association studies (74). With rapidly falling costs, next-generation sequencing of the xMHC appears to be a feasible and efficient strategy for future comprehensive examination of the region in childhood ALL.

It should be noted, however, this ability to test a large number of markers simultaneously, including up to hundreds of high-resolution allelic types for a single HLA locus presents a major concern regarding increased probabilities of false-positive findings due to the multiple tests being conducted. A few available methods for addressing multiple testing include Sidak and Bonferroni corrections, controlling the false-discovery rate, and permutation-based methods (75, 76), but these criteria for defining a statistical significance threshold, in return, put a greater strain on statistical power and sample size requirements. Careful consideration of these requirements, particularly for a rare disease like childhood ALL that appears to benefit from gender-specific and gene-environment interaction evaluation, and the importance of independent replication, suggests the need for coordinated studies and collaboration to fully characterize the role of the xMHC in childhood ALL risk. The Childhood Leukemia International Consortium (CLIC^2^), which currently includes 22 epidemiological studies of childhood leukemia representing 12 countries, was formed in 2005 to facilitate collaborative efforts such as those needed for genetic studies (69). Furthermore, because the extensive LD patterns across the xMHC show population-specificity (70, 77) that vary in the breakdown of conserved sequences, the comparison of multiple homogenous populations of different ancestries can aid in the fine-mapping effort of disease loci (78).

Although the xMHC was one of the first candidate regions examined in childhood ALL over three decades ago, only during these last several years have sufficient advances in understanding of the xMHC been made to permit comprehensive and definitive studies in evaluating its role in childhood ALL. Given the persistent epidemiological support for an infection-related cause, the inconsistent but suggestive evidence of the xMHC and childhood ALL literature, and clear opportunities for application of the most recent knowledge and technologies, we believe that future studies are warranted and have tremendous potential for elucidating the role of this complex region in the genetic susceptibility of childhood ALL.

## Conflict of Interest Statement

The authors declare that the research was conducted in the absence of any commercial or financial relationships that could be construed as a potential conflict of interest.

## Footnotes

^1^http://www.ebi.ac.uk/ipd/imgt/hla

^2^https://ccls.berkeley.edu/clic
